# Range expansion and climatic niche divergence-driven vicariance shaped historical biogeography of Leonticeae (Berberidaceae) across the Northern Hemisphere

**DOI:** 10.1093/aob/mcag150

**Published:** 2026-05-30

**Authors:** Huan-Wen Peng, Lian Lian, Andrey S Erst, Wei Wang

**Affiliations:** State Key Laboratory of Plant Diversity and Specialty Crops, Institute of Botany, Chinese Academy of Sciences, Beijing 100093, China; China National Botanical Garden, Beijing 100093, China; State Key Laboratory of Plant Diversity and Specialty Crops, Institute of Botany, Chinese Academy of Sciences, Beijing 100093, China; China National Botanical Garden, Beijing 100093, China; Central Siberian Botanical Garden, Siberian Branch of Russian Academy of Sciences, Novosibirsk 630090, Russia; State Key Laboratory of Plant Diversity and Specialty Crops, Institute of Botany, Chinese Academy of Sciences, Beijing 100093, China; China National Botanical Garden, Beijing 100093, China; University of Chinese Academy of Sciences, Beijing 100049, China

**Keywords:** Biogeography, climatic niche, disjunct distribution, Neogene, phylogeny, vicariance

## Abstract

**Background and Aims:**

The historical biogeography of the Northern Hemisphere has long been of interest to botanists and biogeographers. Numerous studies have been devoted to investigating the timing, route and direction of dispersals. However, little is known about how the Northern Hemisphere disjunctions formed after range expansion. Here, we used Leonticeae to investigate how biogeographical process and climatic niche divergence shaped the current distribution patterns of Northern Hemisphere plants.

**Methods:**

We used ten DNA loci to reconstruct a phylogeny including ∼79 % species of Leonticeae. Within the phylogenetic framework, we estimated divergence times and reconstructed ancestral ranges and habitats. Furthermore, we conducted phylogenetic principal component analysis on bioclimatic variables and inferred their major shifts and ancestral states.

**Key Results:**

The most recent common ancestor of Leonticeae occupied closed habitats in East Asia and Central Asia at ∼22 Ma. *Caulophyllum* dispersed from East Asia to eastern North America at ∼22–7 Ma, followed by a vicariance event. *Leontice* split from *Gymnospermium* at ∼15 Ma, along with the shift from closed to open habitats. *Gymnospermium* dispersed from East Asia to Central Asia at ∼13–10 Ma, and then independently colonized the East Mediterranean twice during the late Miocene–Pliocene. Marked divergences of climatic niches, especially in precipitation, between the disjunctly distributed sister species/lineages were found.

**Conclusions:**

East Asia serves as a source area for species diversity of the Northern Hemisphere in Leonticeae. Range expansion and subsequent vicariance driven by niche differentiations in precipitation, in conjunction with Neogene geoclimatic events, might have been jointly responsible for the current distribution and endemism of Leonticeae. These findings contribute to our knowledge of the historical biogeography of plants in the Northern Hemisphere.

## INTRODUCTION

Understanding the geographical deployment of organisms through time in the Northern Hemisphere is an important theme in evolutionary biology and ecology (Donoghue *et al.*, 2001; Xiang and Soltis, 2001). One remarkable feature of the Northern Hemisphere biogeography is the intercontinental disjunction of closely related lineages (Raven, 1972). Many temperate plant groups in the Northern Hemisphere exhibit disjunction patterns in two or more of the following regions: East Asia, Central Asia, Europe and North America (Wolfe, 1975; Xiang and Soltis, 2001; Wen *et al.*, 2010). Many studies have investigated the eastern Asian–North American disjunction patterns, especially the classic eastern Asian–eastern North American disjunction (e.g. Wen, 1999; Wen *et al.*, 2010, 2016; Xiang *et al.*, 2018); in recent years, some studies have also been devoted to investigating Eurasian disjunctions (e.g. Manafzadeh *et al.*, 2014; X.-L. Jiang *et al.*, 2019; Manzo and Tomasello, 2025). For other Northern Hemisphere areas, East Asia generally acts as a source of species diversity (D. Jiang *et al.*, 2019; Ling *et al.*, 2024). To better understand the role of East Asia in the formation of intercontinental disjunctions across the Northern Hemisphere, one needs to investigate the historical biogeography of the Northern Hemisphere in a broader phylogenetic context.

Currently, most biogeographic studies have mainly focused on investigation of the timing, route and direction of dispersals responsible for the formation of the disjunctions (e.g. Wen *et al.*, 2010, 2016; Manafzadeh *et al.*, 2014; X.-L. Jiang *et al.*, 2019; Ling *et al.*, 2024; Manzo and Tomasello, 2025; Peng *et al.*, 2025*a*). To date, little is known about how vicariance events happened after range expansion. From the Neogene onwards, the topography and climate in the Northern Hemisphere have undergone tremendous changes (Ruddiman and Kutzbach, 1990; Yin, 2010; Wu *et al.*, 2022); especially, the formation of the three great Asian plateaus, i.e. the Qinghai–Tibet Plateau, the Iran Plateau and the Mongolia Plateau, fundamentally changed the Asian landscapes and climates and thereby affected biotic evolution (Cao *et al.*, 2025). In particular, after the Mid-Miocene Climate Optimum (MMCO, ∼17–14 Ma), Central Asia was dominated by extreme arid environments (Miao *et al.*, 2012; Sun *et al.*, 2015, 2017) owing to global cooling (Westerhold *et al.*, 2020), shrinkage of the Paratethys Sea (Miao *et al.*, 2012) and plateau uplifts (Cao *et al.*, 2025). These geoclimatic events might have caused the occurrence of intercontinental disjunctions in the Northern Hemisphere (e.g. Xiang *et al.*, 2018, 2021). In addition, contemporary climate factors are also responsible for the distribution patterns of plants (Liu *et al.*, 2021; Xia *et al.*, 2022; Wu *et al.*, 2024). Nevertheless, the role of climate factors in shaping disjunct distribution patterns in the Northern Hemisphere is rarely investigated.

**Fig. 1.**
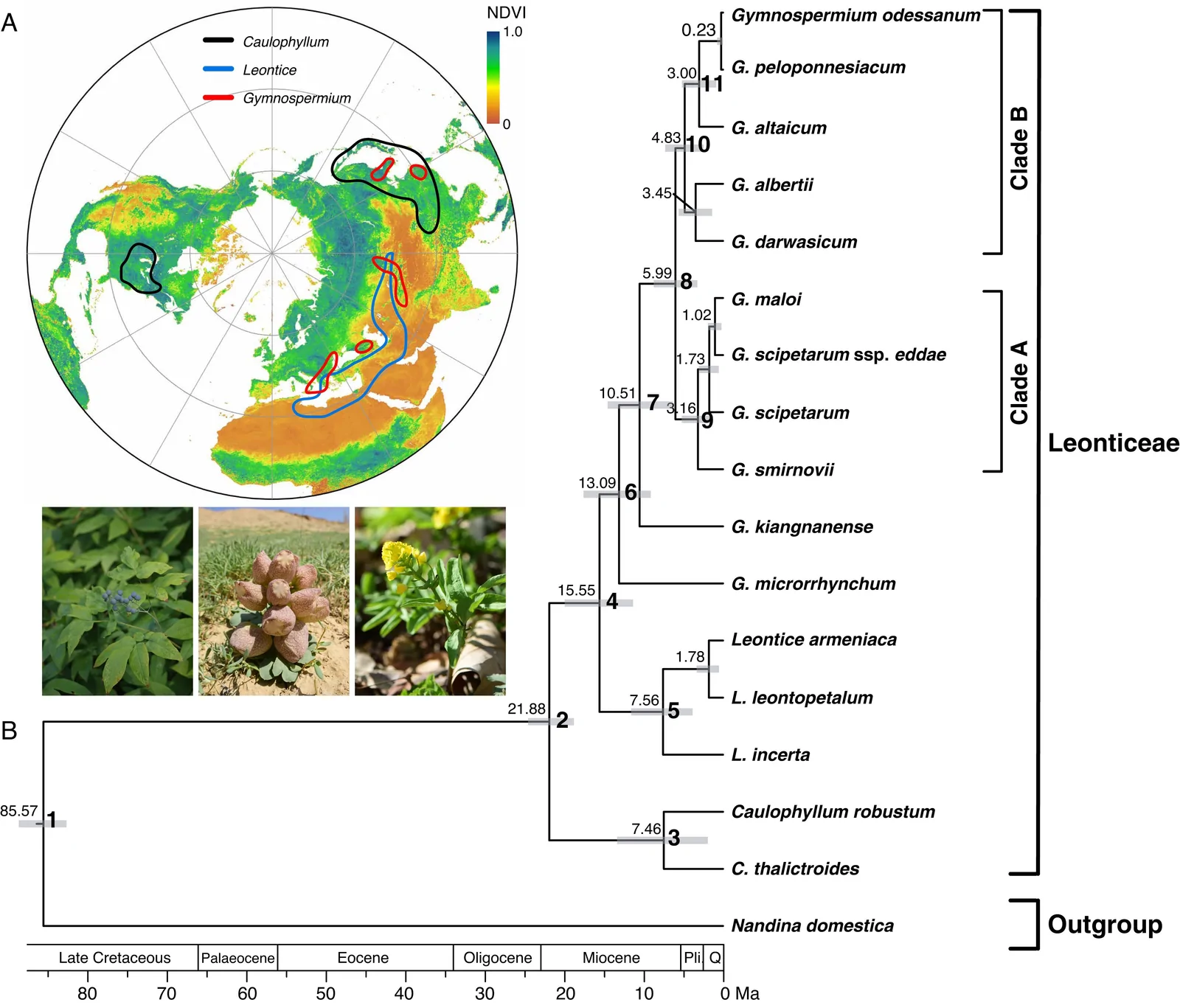
(A) Distribution of Leonticeae on the map of the normalized difference vegetation index (NDVI; obtained from https://iri.columbia.edu/resources/), where higher values represent larger vegetation coverages. Representative plants of Leonticeae are shown at the bottom (from the right: *Caulophyllum robustum*, *Leontice incerta* and *Gymnospermium microrrhynchum*). (B) Chronogram of Leonticeae based on the ten-locus dataset using BEAST. Estimated mean ages are shown at each node with 95 % highest posterior density intervals (grey bars). Nodes of interest are marked 1–11 in bold.

The tribe Leonticeae of the barberry family (Berberidaceae, Ranunculales) is distributed across the north temperate zone and consists of three herbaceous genera and 19 species: *Caulophyllum* (3 spp.), *Gymnospermium* (12 spp.) and *Leontice* (4 spp.) (Fig. 1A; Loconte and Estes, 1989; Loconte, 1993). *Caulophyllum* shows a disjunct distribution between eastern Asia and eastern North America, with two species in eastern North American mesophytic forests and one species in similar East Asian habitats. Members of *Gymnospermium* are understorey components of mesophytic forests, scattered in temperate Eurasia: two species in East Asia, six species in Central Asia and four species in the East Mediterranean. *Leontice* is one of the two desert xerophyte genera in Berberidaceae, with four species distributed from the East Mediterranean to Central Asia. Molecular dating analyses indicate that this tribe began to diversify in the early to mid-Miocene (Wang *et al.*, 2007; Barina *et al.*, 2017; Sun *et al.*, 2018). Thus, Leonticeae provides an ideal model to investigate the Neogene biogeographical process of the Northern Hemisphere and potentially driving factors.

In this study, we first build a robust phylogenic tree for Leonticeae using ten DNA regions from nuclear and plastid genomes. Within the phylogenetic framework, we then estimate divergence times, reconstruct ancestral ranges and habitats, and conduct climatic niche analyses. Our aim is to investigate how the biogeographical process and climatic niche divergence shaped the current distribution pattern of Leonticeae, hence contributing to our knowledge of the historical biogeography of plants in the Northern Hemisphere.

## MATERIALS AND METHODS

### Taxon sampling and data assembly

We sampled 16 taxa representing 15 species (∼79 %) of Leonticeae for phylogenetic analyses, covering all three genera and the geographical range of this tribe (Supplementary Data Table S1). According to the results of broader studies on Berberidaceae (Wang *et al.*, 2007; Sun *et al.*, 2018) or Ranunculales (Peng *et al.*, 2023), we selected *Nandina domestica*, the only species of Nandineae, as the outgroup.

Ten DNA regions, comprising nuclear (26S rDNA, ETS, ITS) and plastid (*ndhA*, *rbcL*, *rpl32-trnL*, *rps16*, *trnG*, *trnL-F*, *trnS-G*) loci, were used in this study. Genomic DNA extraction, PCR amplification and DNA sequencing followed Wang *et al.* (2020). Voucher information and GenBank accession numbers are listed in Supplementary Data Table S1 and the primers used in this study are given in Supplementary Data Table S2. Nucleotide sequences were aligned in Clustal X (Thompson *et al.*, 1997) and then adjusted manually in BioEdit v.5.0.9 (Hall, 1999). Four, four and one difficult-to-align regions in *ndhA* (81 sites), *rpl32-trnL* (83 sites) and *trnS-G* (97 sites), respectively, were excluded from all analyses.

### Phylogenetic analyses

We first employed the maximum likelihood (ML) method to conduct phylogenetic analyses for each individual locus separately. As there was no significant incongruence [≥70 % bootstrap (BS) support] among the three nuclear loci or the seven plastid loci, we then assembled the nrDNA (26S rDNA, ETS, ITS), cpDNA (*ndhA*, *rbcL*, *rpl32-trnL*, *rps16*, *trnG*, *trnL-F*, *trnS-G*) and combined nrDNA and cpDNA datasets. Phylogenetic analyses of these three datasets were carried out using ML and Bayesian inference (BI) methods in RAxML v.7.0.4 (Stamatakis, 2006) and MrBayes v.3.2.5 (Ronquist *et al.*, 2012), respectively. RAxML was run with the fast bootstrap option (1000 replicates) using the GTR + G substitution model for each DNA region. For BI analysis, the best-fit nucleotide substitution model for each DNA region was selected by PartitionFinder v.2.1.1 (Lanfear *et al.*, 2017) based on the corrected Akaike information criterion (AICc). Two independent runs, each comprising four Monte Carlo Markov chains (MCMCs), were conducted by starting from random initial trees and sampling one tree every 1000 generations over two million generations. Stationarity was assessed by the average standard deviation of split frequencies (<0.01) and the effective sample size (ESS) values (>200) using Tracer v.1.7.1 (Rambaut *et al.*, 2018). A majority-rule (>50 %) consensus tree was summarized to calculate posterior probabilities (PPs) for each node after discarding the initial 25 % of sampled trees as burn-in. We adopted the tree-based comparisons of Wiens (1998) and used the thresholds BS ≥ 70 % and PP ≥ 0.95 (Wang *et al.*, 2014) as an indication of strongly supported conflict between the nrDNA and cpDNA datasets. We also assessed cytonuclear discordance using the incongruence length difference (ILD) test (Farris *et al.*, 1994) implemented in PAUP* v.4.0b10 (Swofford, 2003). This test employed 100 replicates, each with 10 random additions using tree bisection–reconstruction branch swapping.

### Divergence time estimation

Because there are no reliable Leonticeae fossils, a two-step strategy was employed to estimate divergence times for this tribe. The first step was to obtain a genus-level chronogram for Berberidaceae via plastid phylogenomic analyses of Ranunculales. A total of 76 taxa were sampled to represent all 17 of the currently recognized genera of Berberidaceae, 33 genera from the other six families of Ranunculales, and 24 genera from 20 other families of basal angiosperms, magnoliids, Chloranthales, monocots, Ceratophyllales and eudicots (Supplementary Data Table S3). Sequences of 76 plastid coding regions were extracted, aligned in Clustal X (Thompson *et al.*, 1997) with manual adjustment in BioEdit v.5.0.9 (Hall, 1999), and finally concatenated into a plastome dataset containing 57 716 characters. The plastome dataset was analysed using ML and BI methods as described above for the multilocus datasets. The penalized likelihood method was applied to calculate the genus-level chronogram for Berberidaceae in treePL v.1.0 (Smith and O’Meara, 2012) using the optimal ML tree and 1000 bootstrap replicates with branch lengths generated by RAxML. Twelve fossil calibration points were used to constrain the minimum bound of node ages (Supplementary Data Fig. S1, Supplementary Data Table S4). To avoid overestimation of root age, we set a maximum age of 198.1 Ma for the root, which is the estimated stem group age of Nymphaeales (Li *et al.*, 2019 ). Cross-validation was run in treePL to test seven values for the smoothing parameter (0.001, 0.01, 0.1, 1, 10, 100 and 1000), and resulted in an optimal smoothing parameter of 0.1. Confidence intervals for ages of each node were calculated from 1000 bootstrap replicates using TreeAnnotator v.2.3.0 in the BEAST package (Bouckaert *et al.*, 2014).

The second step was to estimate species-level divergence times for Leonticeae based on the ten-locus dataset in BEAST v.2.3.0 (Bouckaert *et al.*, 2014). The obtained stem (86.06 Ma) and crown (22.36 Ma) group ages of Leonticeae in treePL were used as the two secondary calibration points with normal prior distributions giving their 95 % confidence intervals (standard deviation = 1.2). The BEAST dating analysis was conducted under the uncorrelated relaxed log-normal clock model, the birth–death tree prior process, the GTR + G model for each DNA region separately, and with 80 million generations and trees sampled every 10 000 generations. Convergence and ESS values (>200) were checked using Tracer v.1.7.1 (Rambaut *et al.*, 2018). The maximum clade credibility (MCC) tree with the mean age and 95 % highest posterior density (HPD) for each node was calculated using TreeAnnotator with the first 25 % of samples as burn-in.

### Ancestral range estimation

Ancestral range estimation was performed using the R package BioGeoBEARS v.1.1.1 (Matzke, 2013) on the MCC tree from the BEAST analysis. Based on the floristic divisions of Takhtajan (1986) and present-day distributions of Leonticeae species (Fig. 1A; Loconte and Estes, 1989; Loconte, 1993; Barina *et al.*, 2017; Rosati *et al.*, 2019), we defined four biogeographical regions: East Asia, Central Asia (including the Caucasus, Irano-Anatolian), East Mediterranean (including the north of the Black Sea) and eastern North America. The maximum number of ancestral ranges was limited to two because no extant species occurs in more than two regions. Dispersal probabilities between pairs of regions were specified for three separate time slices (Supplementary Data Table S5) according to known geological events (McKenna, 1983; Rögl, 1999; Marincovich and Gladenkov, 2001; Gladenkov *et al.*, 2002). The dispersal probability was set to 1.0 for adjacent areas, 0.5 for areas separated by one area or intermittent barriers (e.g. land bridge and seaway), 0.25 for areas separated by two areas, and 0.1 for areas separated by a large oceanic barrier. As the jump-dispersal (*j*) parameter remains controversial (Ree and Sanmartín, 2018; Matzke, 2022), we only tested two biogeographical models: DEC (dispersal–extinction–cladogenesis; Ree and Smith, 2008) and DIVALIKE (a likelihood implementation of dispersal–vicariance analysis;Ronquist, 1997). We did not test the BAYAREALIKE (a likelihood implementation of Bayesian inference of historical biogeography for discrete areas;Landis *et al.*, 2013) model because it does not allow vicariance-driven events (Matzke, 2014). The fit for different models was assessed using the AICc.

### Ancestral habitat reconstruction

Ancestral habitat reconstruction was conducted using ML and BI methods. Information on habitats was compiled from the literature (Loconte and Estes, 1989; Loconte, 1993), herbarium specimens and field observation. Two habitat types were categorized based on light requirements: closed (forest understorey with at least a partial canopy) and open (without a canopy formed by other plants). The ML analysis was run in Mesquite v.3.81 (Maddison and Maddison, 2021) using the Markov *k*-state one-parameter model and the Trace Character History option. For the BI analysis, a set of 1000 trees randomly sampled from phylogenetic trees via MrBayes were analysed using the MCMC method with the MultiState model (Pagel *et al.*, 2004) in BayesTraits (Meade and Pagel, 2018), implemented in RASP v.4.2 (Yu *et al.*, 2020). The MCMC of BayesTraits was run for five million generations, discarding the first 10 % samples as burn-in, and the results were summarized on the MCC tree.

### Climatic niche analyses

Georeferenced specimen occurrence data for the 16 sampled taxa of Leonticeae were collected from the Global Biodiversity Information Facility (GBIF; https://www.gbif.org) using the occ_search function in the R package rgibf v.1.4.0 (Chamberlain and Boettiger, 2017), the National Plant Specimen Resource Center (http://www.cvh.ac.cn) and published papers (Barina *et al.*, 2017; Rosati *et al.*, 2019). We removed records where coordinates were duplicated, overseas, outside the native range of each taxon, or the same for latitude and longitude. In addition, to correct the bias owing to dense collections of specimens in some regions, we retained only one valid record within each 30 arc-second pixel. Finally, a total of 1439 georeferenced records were retained. For each record, we extracted 19 bioclimatic variables from the 30 arc-second resolution data layers for 1970–2000 downloaded from WorldClim v.2.1 (Fick and Hijmans, 2017) using ArcGIS v.10.6 (ESRI, Redlands, CA, USA). We then calculated the species mean value of each of the 19 variables.

To explore the potential climatic factors that drove the biogeographical patterns of Leonticeae, we employed an approach similar to a phylomorphospace (Sidlauskas, 2008) on phylogenetic principal component analysis (pPCA; Revell, 2009) following Li *et al.* (2020). The pPCA was performed on the Leonticeae phylogeny with the bioclimatic variable dataset in the R package phytools v.0.7.47 (Revell, 2012). The first two principal components (pPC1 and pPC2) explained more variation than expected based on a broken-stick distribution for a total of 97.1 % and thus were used in the phylomorphospace analysis.

To evaluate climatic niche shifts in Leonticeae, we used a phylogenetic lasso method to detect evolutionary shifts in the optima of the five variables mostly related to pPC1 and pPC2 under an Ornstein–Uhlenbeck process in the R package l1ou v.1.41 (Khabbazian *et al.*, 2016). The phylogenetic Bayesian information criterion was applied to select the best model and to estimate shift configuration, which can account for both phylogenetic correlation between species and the complexity of estimating an unknown number of shifts at unknown locations in the phylogeny. Bootstrap support was evaluated for the estimated niche shifts with 1000 replicates. To further infer evolutionary patterns of bioclimatic variables with the detected niche shifts, we reconstructed their ancestral states using fast ML estimation in the R package phytools v.0.7.47 (Revell, 2012). We also used *t*-tests to compare differences of these bioclimatic variables among species, and visualized their distribution using boxplots in ggplot2 v.3.3.2 (Wickham, 2016).

## RESULTS

### Phylogeny

The ML and BI analyses of the plastome dataset generated identical and highly resolved phylogenetic trees for Ranunculales with strong support for most nodes (Supplementary Data Fig. S2). Ranunculales and its seven families were all recognized as monophyletic (BS = 100 %, PP = 1.0). Within Ranunculales, Eupteleaceae is basalmost, followed by Papaveraceae; and Circaeasteraceae-Lardizabalaceae, Menispermaceae and Ranunculaceae are successive sister taxa to Berberidaceae. All the three subfamilies of Berberidaceae (Berberidoideae, Nandinoideae and Podophylloideae) are strongly supported as monophyletic. Within Nandinoideae, the monophyly of Leonticeae is strongly supported, sister to *Nandina* (BS = 100 %, PP = 1.0).

The aligned nrDNA dataset contained 2485 nucleotides (26S rDNA, 1329 bp; ETS, 519 bp; ITS, 637 bp). The aligned cpDNA included 7213 characters (*ndhA*, 1122 bp; *rbcL*, 1395 bp; *rpl32-trnL*, 1267 bp; *rps16*, 905 bp; *trnG*, 746 bp; *trnL-F*, 989 bp; *trnS-G*, 789 bp). Tree-based comparisons indicated that the topology of Leonticeae phylogeny generated based on the nrDNA dataset was highly congruent with the cpDNA tree (Supplementary Data Fig. S3). The ILD test generated a *P*-value of 0.17, further supporting the idea that that the nrDNA and cpDNA datasets are not significantly incongruent. Thus, the nrDNA and cpDNA datasets were combined into a single dataset that comprised 9698 characters. The ML and BI analyses yielded identical topologies with ∼93 % of nodes receiving high support (BS ≥ 70 % and PP ≥ 0.95; Supplementary Data Fig. S4). All three genera of Leonticeae are strongly supported as monophyletic. *Caulophyllum* is sister to the clade containing *Leontice* and *Gymnospermium* (BS = 100 %, PP = 1.0). Within *Leontice*, *L. incerta* is sister to other species (BS = 100 %, PP = 1.0). Within *Gymnospermium*, *G. microrrhynchum* is the earliest-diverging lineage (BS = 90 %, PP = 1.0), followed by *G. kiangnanense*; other *Gymnospermium* species formed two clades (A and B). In Clade A, *G. smirnovii* is sister to other taxa (BS = 99 %, PP = 1.0), and *G. maloi* is nested in *G. scipetarum* (BS = 93 %, PP = 1.0). In Clade B, *G. albertii* and *G. darwasicum* formed a clade (BS = 96 %, PP = 0.99), and *G. altaicum* is sister to the clade containing *G. odessanum* and *G. peloponnesiacum* (BS = 92 %, PP = 0.99).

### Divergence time estimation and biogeographical reconstruction

**Fig. 2.**
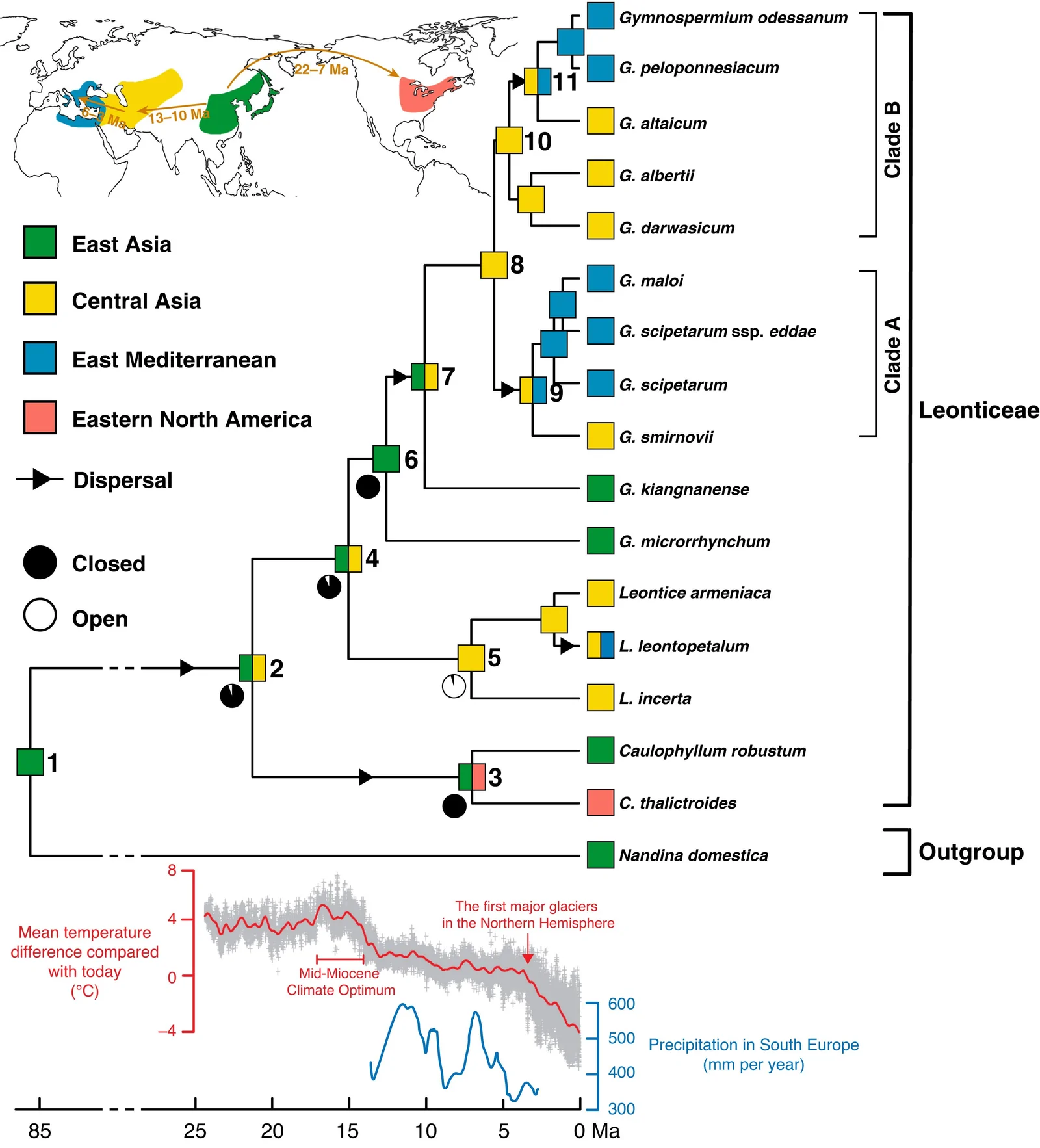
Ancestral range and habitat reconstructions for Leonticeae. The most likely ancestral range estimated using BioGeoBEARS under the DIVALIKE model is presented at each node as a coloured square (see Supplementary Data Fig. S5 for details). Pie charts close to nodes show the relative probabilities of alternative ancestral habitats inferred in Mesquite (see Supplementary Data Fig. S6 for details). Numbers in bold near branches indicate the node number, as referred to in Fig. 1. The curve of mean temperature difference with respect to today is modified from Westerhold *et al.* (2020). The precipitation graph for South Europe is modified from van Dam (2006).

The chronogram based on the plastome dataset is shown in Supplementary Data Fig. S1. The crown group ages of Ranunculales and Berberidaceae were estimated at 135.97 Ma (95 % HPD: 134.50–137.61) and 104.03 Ma (95 % HPD: 102.46–104.96), respectively. The stem and crown group ages of Leonticeae were estimated at 86.06 Ma (95 % HPD: 83.85–88.41) and 22.36 Ma (95 % HPD: 19.94–24.60), respectively. The species-level age estimation for Leonticeae based on the ten-locus dataset is displayed in Fig. 1B. For biogeographical analyses, the DIVALIKE model (AICc = 56.80) outperformed the DEC model (AICc = 60.69), so the results under the DIVALIKE model are reported here (Fig. 2, Supplementary Data Fig. S5). Leonticeae originated in East Asia at 85.57 Ma (95 % HPD: 82.68–88.68; node 1) and dispersed into Central Asia at 21.88 Ma (95 % HPD: 18.79–24.55; node 2). In *Caulophyllum*, East Asian *C. robustum* split from North American *C. thalictroides* at 7.46 Ma (95 % HPD: 1.91–13.34; node 3). *Leontice* split from *Gymnospermium* at 15.55 Ma (95 % HPD: 11.34–19.92; node 4) and became differentiated in Central Asia at 7.56 Ma (95 % HPD: 3.84–11.58; node 5). *Gymnospermium* started to diversify in East Asia at 13.09 Ma (95 % HPD: 9.11–17.57; node 6) and then dispersed into Central Asia at 10.51 Ma (95 %HPD: 6.36–14.53; node 7). Clades A and B separated at 5.99 Ma (95 % HPD: 3.24–8.73; node 8) and independently dispersed from Central Asia into the East Mediterranean at 3.16 Ma (95 %HPD: 1.28–5.21; node 9) and 3.00 Ma (95 % HPD: 0.85–5.17; node 11), respectively.

### Ancestral habitat and climatic niche analyses

**Fig. 3.**
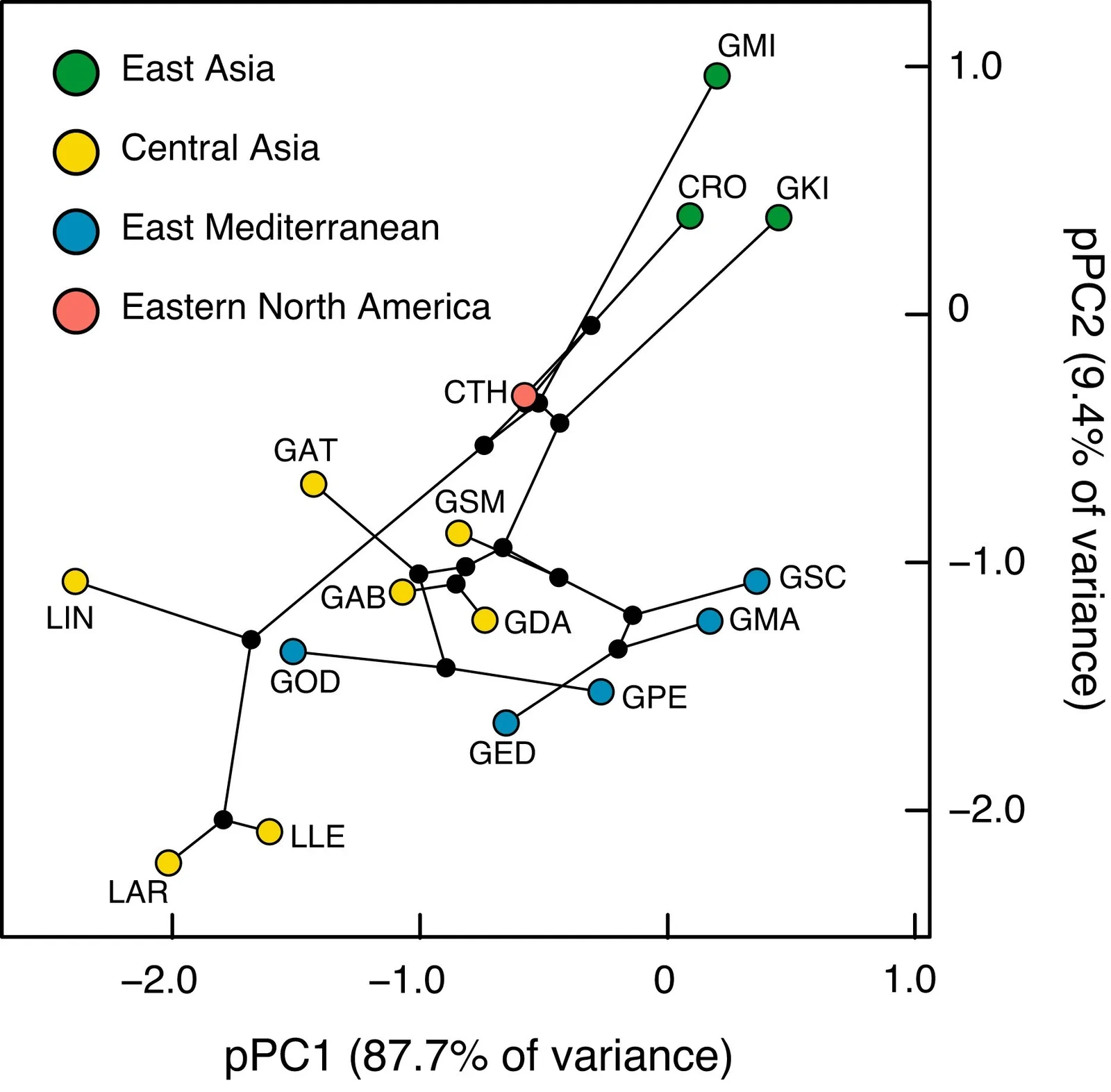
Phylomorphospace on phylogenetic principal component analysis axes and biogeographical regions of Leonticeae. The pPC1 axis represents differences in annual precipitation, precipitation of the wettest month/quarter, and precipitation of the coldest quarter; the pPC2 axis represents differences in precipitation of the warmest quarter. CRO, *C. robustum*; CTH, *C. thalictroides*; GAB, *G. albertii*; GAT, *G. altaicum*; GDA, *G. darwasicum*; GKI, *G. kiangnanense*; GMI, *G. microrrhynchum*; GOD, *G. odessanum*; GPE, *G. peloponnesiacum*; GSC, *G. scipetarum*; GMA, *G. maloi*; GED, *G. scipetarum* ssp. *eddae*; GSM, *G. smirnovii*; LAR, *L. armeniaca*; LIN, *L. incerta*; LLE, *L. leontopetalum*.

**Fig. 4.**
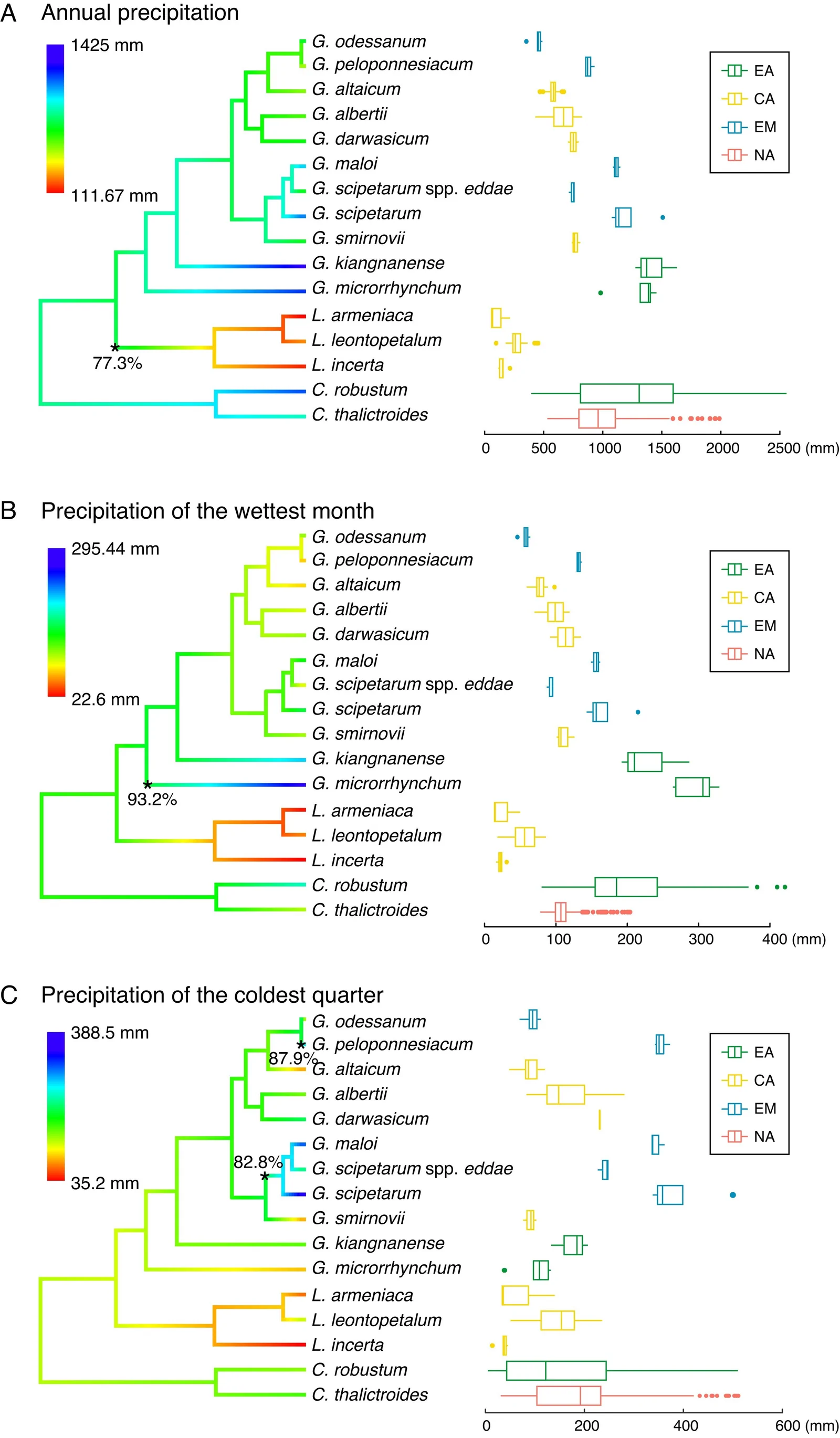
Evolutionary patterns of annual precipitation (A), precipitation of the wettest month (B) and precipitation of the coldest quarter (C) in Leonticeae. Asterisks indicate predicted niche shifts with bootstrap values. Box plots denote the distribution of bioclimatic variables of each species. EA, East Asia; CA, Central Asia; EM, East Mediterranean; NA, eastern North America.

Ancestral habitat reconstructions using ML and BI approaches generated identical results (Fig. 2, Supplementary Data Fig. S6). Closed habitat is the ancestral state in Leonticeae and a shift from closed to open habitat occurred in *Leontice*. Based on the loadings of bioclimatic variables, pPC1 mainly described annual precipitation, precipitation of the wettest month/quarter and precipitation of the coldest quarter, and pPC2 captured precipitation of the warmest quarter (Supplementary Data Table S6). Lower values of pPC1 and pPC2 were mostly related to lower precipitation. The phylomorphospace projection shows that *Leontice* adapts to environments with lower precipitation compared with *Caulophyllum* and *Gymnospermium* (Fig. 3). In *Caulophyllum*, East Asian *C. robustum* inhabits localities with more abundant rainfall than eastern North American *C. thalictroides*. In *Gymnospermium*, 11 species are clustered by biogeographical regions. Among the five variables mostly related to pPC1 and pPC2, we identified four niche shifts in three variables: one in annual precipitation (BS = 77.3 %; Fig. 4A), one in precipitation of the wettest month (BS = 93.2 %; Fig. 4B) and two in precipitation of the coldest quarter (BS = 82.8% and 87.9 %; Fig. 4C). Significant differences (*P* < 0.05) of these three variables among most species were also found (Supplementary Data Table S7).

## DISCUSSION

### Phylogeny and divergence times

Based on the plastome dataset, inter-familial relationships within Ranunculales were well resolved (Supplementary Data Fig. S2) and in line with previous studies (e.g. Wang *et al.*, 2009; Hoot *et al.*, 2015; Peng *et al.*, 2023; Zuntini *et al.*, 2024). Within Berberidaceae, three major clades were recognized, corresponding to Podophylloideae, Berberidoideae and Nandinoideae. In Nandinoideae, *Nandina* is sister to the monophyletic Leonticeae. Both the plastome and ten-locus datasets recovered *Caulophyllum* as the sister group of the *Gymnospermium–Leontice* clade (Supplementary Data Figs S2 and S4). These results are consistent with previous studies using multiple DNA regions (Wang *et al.*, 2007, 2009) or plastid genomic data (Sun *et al.*, 2018; Hsieh *et al.*, 2022; Song *et al.*, 2022). Within *Gymnospermium*, *G. microrrhynchum* is sister to all other species (BS = 90 %, PP = 1.00), followed by *G. kiangnanense*, which is in agreement with the result of Song *et al.* (2022). The interspecific relationships within Clade A and Clade B are congruent with the results of Barina *et al.* (2017) and Rosati *et al.* (2019), but some nodes in our phylogeny using more DNA loci received higher support values.

Our treePL analysis based on the plastome dataset suggests a crown group age of 135.97 Ma (95 % HPD: 134.50–137.61) for Ranunculales (Supplementary Data Fig. S1), in agreement with the results of Ramírez-Barahona *et al.* (2020; 138.5 Ma, 95 % HPD 127.47–148.68). We suggest a crown group age of 104.03 Ma (95 % HPD: 102.46–104.96) for Berberidaceae and a stem group age of 86.06 Ma (95 % HPD: 83.85–88.41) for Leonticeae (Supplementary Data Fig. S1), which are much older than those estimated by Sun *et al.* (2018) and Hsieh *et al.* (2022). Six ranunculid fossils were used as calibration points in our study, while only two and three ranunculid fossils were used in Sun *et al.* (2018) and Hsieh *et al.* (2022), respectively. Sun *et al.* (2018) constrained the Berberidaceae crown group age to 33.9 Ma based on the fossils assigned to *Mahonia* (Schorn, 1966; Manchester, 1999), which obviously underestimated the ages of Berberidaceae because an older leaf fossil *Mahonia* sp. from the John Day Formation, Oregon, USA at ∼38.8 Ma (Manchester, 2000) was a stem group member of *Mahonia* (Chen *et al.*, 2020). We used *Mahonia* sp. to constrain the stem group age of *Mahonia* (Supplementary Data Fig. S1, Supplementary Data Table S4) following Chen *et al.* (2020). Hsieh *et al.* (2022) used the endocarp fossil *Prototinomiscium vangerowii* from the Turonian of Central Europe (Knobloch and Mai, 1986) to constrain the stem group age of Menispermaceae, but phylogenetic analysis supported this fossil as a crown group member of Menispermaceae (Jacques *et al.*, 2011), as used in this study. Our BEAST analysis based on the ten-locus dataset indicates that Leonticeae began to diversify at 21.88 Ma (95 %HPD: 18.79–24.55; node 2) and the split of *Leontice* and *Gymnospermium* was at 15.55 Ma (95 %HPD: 11.34–19.92; node 4), which are highly consistent with the results of Barina *et al.* (2017) and Hsieh *et al.* (2022).

### Genus-level biogeography of Leonticeae

Our molecular dating and biogeographical analyses show that Leonticeae originated in East Asia in the Lower Cretaceous (∼86 Ma; node 1) and dispersed into Central Asia in the early Miocene (∼22 Ma; node 2, Fig. 2). The long branch between the stem and crown groups of Leonticeae implies that this tribe might be a relic but recently thriving lineage, like *Oreomecon* (Papaveraceae; Peng *et al.*, 2025b). A vicariance event occurred between East Asian *Gymnospermium* and Central Asian *Leontice* at ∼15 Ma (node 4, Fig. 2), temporally congruent with the broad-scale uplifts of the Iranian Plateau and the Qinghai–Tibet Plateau and adjacent mountains (Mouthereau, 2011; Mouthereau *et al.*, 2012; Xiang *et al.*, 2021; Cao *et al.*, 2025). Mountain building, together with a drop in global temperature after the MMCO (Fig. 2; Westerhold *et al.*, 2020), resulted in significant shrinkage of the Paratethys Sea, and thereby intensified the aridification of Central Asia (Miao *et al.*, 2012; Sun *et al.*, 2017; Tang *et al.*, 2023). The phylomorphospace projection of 19 bioclimatic variables supports the idea that *Leontice* adapts to environments with lower rainfalls than *Gymnospermium* (Fig. 3), and ancestral state reconstruction for annual precipitation indicates a niche shift (BS = 77.3 %) towards lower precipitation in *Leontice* (Fig. 4A), with significant differences between *Leontice* and two other genera of Leonticeae (Supplementary Data Table S7). In addition, our habitat analyses suggest a shift from closed to open habitat in *Leontice* (Fig. 2, Supplementary Data Fig. S6). Thus, ecological divergence resulting from the intensified aridification in Central Asia might have driven the split between Central Asian xeric *Leontice* and its East Asian sister group. The fruits of *Leontice* are bladder-like and inflated and its seeds are thus adapted for dispersal as tumbleweeds (Loconte, 1993), which might have enabled subsequent range expansion and diversification of *Leontice* in open dry habitats of Central Asia.

### Eastern Asian–eastern North American disjunct distribution in *Caulophyllum*

*Caulophyllum* is disjunctly distributed in the temperate zone of East Asia and eastern North America (Fig. 1A). Our ancestral range reconstruction indicates that this genus migrated from East Asia to eastern North America ∼22–7 Ma (between nodes 2 and 3, Fig. 2), supporting the out-of-Asia hypothesis (D. Jiang *et al.*, 2019; Ling *et al.*, 2024). Given that *C. robustum* is mainly distributed in north-eastern Asia, the Bering Land Bridge connecting the land of Asia and North America (Marincovich and Gladenkov, 2001; Gladenkov *et al.*, 2002) may have provided an available corridor for the range expansion of *Caulophyllum* from East Asia to eastern North America during the Miocene, as in many other temperate plant groups (Donoghue and Smith, 2004; Wen *et al.*, 2010; Huang *et al.*, 2025).

After migrating into eastern North America, a vicariance event took place in the late Miocene (∼7 Ma), resulting in the emergence of the eastern Asian–eastern North American disjunction between *C. robustum* and *C. thalictroides*. Both *C. robustum* and *C. thalictroides* grow in temperate mesophytic forests, but our climatic niche analysis shows that climatic niches diverged between them: East Asian *C. robustum* inhabits environments with higher precipitation compared with eastern North American *C. thalictroides* (Fig. 3, Supplementary Data Table S7). By employing climate models integrated with climate proxies, He *et al.* (2025) indicated that during the Miocene the spring persistent rainfall in East Asia became more abundant than that in North America. The Appalachian Mountains in eastern North America might also prevent moisture of the Atlantic Ocean from reaching inland to some extent, which resulted in lower precipitation compared with East Asia at the same latitudes (Fick and Hijmans, 2017). Taking these findings together, niche differentiation in precipitation possibly drove the vicariance event that is responsible for the formation of the eastern Asian–eastern North American disjunction in *Caulophyllum*. As reviewed in Wen *et al.* (2010), most temperate disjunct lineages between eastern Asia and eastern North America diverged during the Miocene. Whether climatic niche divergence played important roles in shaping the eastern Asian–eastern North American disjunctions of other taxa needs to be tested in the future.

### Eurasian disjunct distributions in *Gymnospermium*

*Gymnospermium* has a scattered distribution in temperate mesophytic forests in Eurasia (Fig. 1A). Our molecular dating and biogeographical analyses show that *Gymnospermium* started to diversify in East Asia at ∼13 Ma (node 6, Fig. 2). Both the first two early-diverging species are distributed in East Asia: *G. microrrhynchum* in northeastern Asia and *G. kiangnanense* in southeastern China (Ying *et al.*, 2011). Our climatic niche analyses show that the two species prefer moister conditions, especially with more summer precipitation (Figs 3 and 4A, B). The humid northern and southern regions of East Asia were thought to be divided by an east–west-orientated arid belt between 35°N and 45°N in most of the Palaeogene (Guo *et al.*, 2008), hindering floristic exchanges between the two regions (Milne, 2006). Owing to the intensification of the East Asian summer monsoon, the intensity of this arid belt decreased in the early to mid-Miocene (∼24–11 Ma) (Tiffney and Manchester, 2001; Guo *et al.*, 2008), which might have allowed the population exchange of *Gymnospermium* between the northern and southern East Asian regions. The original ages of these two East Asian *Gymnospermium* species are between 13 and 10 Ma (nodes 6 and 7, Fig. 2). The north–south divergence in Asian butternuts also occurred with similar timing (Bai *et al.*, 2016). During this period, the east–west arid belt redeveloped (∼11–5.3 Ma; Guo *et al.*, 2008), which could have acted as an ecological barrier for the exchange of *Gymnospermium* and other East Asian mesic plants between northern and southern East Asian regions.

*Gymnospermium* dispersed from East Asia into Central Asia, followed by a vicariance event between these two regions at ∼10 Ma (node 7, Fig. 2), which is temporally in agreement with the northward propagation of the north-eastern Pamir mountains (Thompson *et al.*, 2015) and the reactivated uplift of the Tianshan (Manafzadeh *et al.*, 2017), especially the southern Tianshan (Zhang and Sun, 2011). The collision between the Pamir and Tianshan gradually obstructed the moisture channel into the Tarim Basin, contributing to an extreme arid climate in the Tarim Basin (Sun and Liu, 2006; Liu *et al.*, 2014; Sun *et al.*, 2017). The phylomorphospace projection plot indicates that, in *Gymnospermium*, Central Asian species inhabit relatively drier environments than East Asian species (Fig. 3). The drier and cooler biomes expanded in Central Asia during the late Miocene (Pound *et al.*, 2012). Thus, the dramatic aridification of northwestern China might have caused climatic niche divergence between Central Asian and East Asian *Gymnospermium* and thereby resulted in the vicariance between the two regions.

During the latest Miocene to Pliocene, two independent migration events from Central Asia to East Mediterranean occurred in Clade A and Clade B, respectively (∼6–3 Ma; nodes 8–9 and 10–11, Fig. 2). Given that *Gymnospermium* inhabits closed habitats (Supplementary Data Fig. S6), the continuous forests of central Anatolia that occurred 7.2–5.3 Ma (Kayseri-Özer, 2017) might have provided a dispersal corridor for the two westward migrations of this genus. Then, two vicariance events between Central Asian and East Mediterranean lineages happened almost simultaneously ∼3 Ma (nodes 9 and 11), temporally consistent with the intensified aridification of the Anatolian Plateau in the late Pliocene (Kayseri-Özer, 2017; Meijers *et al.*, 2020) and the first major glacier event in the Northern Hemisphere (Fig. 2; Westerhold *et al.*, 2020). The aridification and Plio-Pleistocene glacier fluctuations might have disrupted the forest belt and hampered population exchange of *Gymnospermium* between Central Asia and East Mediterranean, as found in *Epimedium* (Zhang *et al.*, 2007) and land snails (*Oxychilus*; Neiber *et al.*, 2022). Compared with Central Asian species, most East Mediterranean *Gymnospermium* species inhabit environments with more precipitation, especially annual and winter precipitation (higher values of pCP1; Fig. 3, Supplementary Data Table S7). Meanwhile, we also identified a major niche shift (BS = 82.8 %) towards more winter precipitation in the clade composed of East Mediterranean *G. scipetarum*, *G*. *scipetarum* ssp. *eddae* and *G*. *maloi* (Fig. 4C). Using small mammals as palaeoprecipitation proxies, van Dam (2006) also revealed increased annual and winter precipitation in South Europe 3.3–2.5 Ma (Fig. 2). Thus, our data suggest that climatic niche divergence might also play an important role in driving the vicariance events between Central Asia and East Mediterranean *Gymnospermium* lineages.

### Conclusions

Our study presents a comprehensive phylogeny for Leonticeae based on ten DNA regions from nuclear and plastid genomes. We show that Leonticeae originated in the Upper Cretaceous, but did not diversify until the early Miocene, implying that this tribe is a relic but recently thriving lineage. Within Leonticeae, we inferred two dispersal routes: one is from East Asia to eastern North America and the other is from East Asia to Central Asia and westwards to East Mediterranean, suggesting that East Asia is an important source of biodiversity for other Northern Hemisphere temperate regions. Five vicariance events were inferred in Leonticeae, which resulted in the current disjunct distribution patterns of the tribe across the Northern Hemisphere. Marked divergence of climatic niches, especially related to precipitation variables, were found between the disjunct lineages in Leonticeae, which, together with geoclimatic events, might have been responsible for the eastern Asian–eastern North American and Eurasian disjunction patterns of Leonticeae. Our findings suggest that the Neogene biodiversity of the Northern Hemisphere might have been jointly shaped by both range expansion and subsequent vicariance driven by climatic niche divergence among closely related lineages.

## Supplementary Material

mcag150_Supplementary_Data

## SUPPLEMENTARY DATA

Supplementary data are available at *Annals of Botany* online and consist of the following. Table S1: taxon, voucher identification and GenBank accession numbers for the sequences used in this study. Table S2: primers used for amplification and sequencing in this study. Table S3: taxon and GenBank accession numbers for plastid phylogenomic analyses of Ranunculales in this study. Table S4: fossil calibration points as minimum age constraints used in this study. Table S5: dispersal multiplier matrix. Table S6: principal component loadings of each bioclimatic variable from the phylogenetic principal component analysis. Table S7: matrix of *P*-values from pairwise significance tests among species for annual precipitation, precipitation of the wettest month and precipitation of the coldest quarter. Figure S1: time-calibrated tree of Ranunculales inferred from the plastome dataset using TreePL. Figure S2: ML tree of Ranunculales inferred from the plastome dataset. Figure S3: comparison of topologies for Leonticeae obtained from the nrDNA and cpDNA datasets. Figure S4: ML tree of Leonticeae inferred from the ten-locus dataset. Figure S5: ancestral area reconstructions using BioGeoBEARS under the DEC (top) and DIVALIKE (bottom) models. Figure S6: ancestral habitat reconstructions using the maximum likelihood method in Mesquite (left) and using Bayesian inference in BayesTraits (right).
