# Supplementary material for: Range expansion and climatic niche divergence-driven vicariance shaped historical biogeography of Leonticeae (Berberidaceae) across the Northern Hemisphere
Source: Ann Bot. 2026 May 30;138(4):1182–93. doi: 10.1093/aob/mcag150 (PMC13595243; doi:10.1093/aob/mcag150)
Supplement: Supplementary file 1 [file mcag150_supplementary_data.doc]

Supplementary data

Fig. S1. Time-calibrated tree of Ranunculales inferred from the plastome dataset using TreePL.

Fig. S2. ML tree of Ranunculales inferred from the plastome dataset.

Fig. S3. Comparison of topologies for Leonticeae obtained from the nrDNA and cpDNA datasets.

Fig. S4. ML tree of Leonticeae inferred from the ten-locus dataset.

Fig. S5. Ancestral area reconstructions using BioGeoBEARS under the DEC (top) and DIVALIKE (bottom) models.

Fig. S6. Ancestral habitat reconstructions using maximum likelihood method in Mesquite (left) and using Bayesian inference in BayesTraits (right).

Table S1. Taxon, voucher identification and GenBank accession numbers for the sequences used in this study.

Table S2. Primers used for amplification and sequencing in this study.

Table S3. Taxon and GenBank accession numbers for plastid phylogenomic analyses of Ranunculales in this study.

Table S4. Fossil calibration points as minimum age constraints used in this study.

Table S5. Dispersal multiplier matrix.

Table S6. Principal component loadings of each bioclimatic variable from the phylogenetic principal component analysis.

Table S7. Matrix of *p*-values from pairwise significance tests among species for annual precipitation, precipitation of wettest month, and precipitation of coldest quarter.

Fig. S1. Time-calibrated tree of Ranunculales inferred from the plastome dataset using TreePL. Estimated mean ages are shown at each node with 95% highest posterior density intervals (grey bars). Red pentagrams represent fossil calibration points, nodes A–L, as referred to Table S4.

Fig. S2. ML tree of Ranunculales inferred from the plastome dataset. Numbers above branches are bootstrap (BS) values and Bayesian posterior probabilities (PP). An asterisk (*) indicates BS = 100% or PP = 1.00.

Fig. S3. Comparison of topologies for Leonticeae obtained from the nrDNA and cpDNA datasets. Numbers above branches are bootstrap (BS) percentages and Bayesian posterior probabilities (PP). Only nodes with BS ≥ 70% and PP ≥ 0.95 were indicated.

Fig. S4. ML tree of Leonticeae inferred from the ten-locus dataset. Numbers above branches are bootstrap (BS) values and posterior probabilities (PP). An asterisk (*) indicates BS = 100% or PP = 1.00.

Fig. S5. Ancestral area reconstructions using BioGeoBEARS under the DEC (top) and DIVALIKE (bottom) models. Pie charts on each node show the relative probabilities of alternative ancestral distributions.

Fig. S6. Ancestral habitat reconstructions using maximum likelihood method in Mesquite (left) and using Bayesian inference in BayesTraits (right).

Table S1. *Taxon, voucher identification and GenBank accession numbers for the sequences used in this study*

| Taxon | Voucher | ETS | ITS | 26S | *ndhA* | *rbcL* | *rpl32-trnL* | *rps16* | *trnG* | *trnL-F* | *trnS-G* |
| --- | --- | --- | --- | --- | --- | --- | --- | --- | --- | --- | --- |
| ***Caulophyllum* Michx.** |  |  |  |  |  |  |  |  |  |  |  |
| *C. robustum* Maxim. | Chen et al. 960773 (PE) | PZ336760 | PZ298204 | FJ626455 | PZ336769 | PZ336725 | PZ336778 | PZ336732 | PZ336787 | PZ336741 | PZ336750 |
| *C. thalictroides* (L.) Michx. |  |  | L77158/ L77159 | AF389240 |  | AF190442 |  |  |  | FJ626552 |  |
| ***Gymnospermium* Spach** |  |  |  |  |  |  |  |  |  |  |  |
| *G. albertii* (Regel) Takht. | Pimenov et al. *s.n.* (MW) | PZ336761 | KX257208 |  | PZ336770 | PZ336726 |  | PZ336733 | PZ336788 | PZ336742 | PZ336751 |
| *G. altaicum* (Pall.) Spach | SRAE 2004250a (PE) | PZ336762 | PZ298205 |  | PZ336771 |  | PZ336779 | PZ336734 | PZ336789 | PZ336743 | PZ336752 |
| *G. darwasicum* (Regel) Takht. |  |  | KX257209 |  |  |  |  |  |  | KX272779 |  |
| *G. kiangnanense* (P.L.Chiu) Loconte | Zhang J 0001 (PE) | PZ336763 | PZ298206 | PZ298196 | PZ336772 | PZ336727 | PZ336780 | PZ336735 | PZ336790 | PZ336744 | PZ336753 |
| *G. microrrhynchum* (S.Moore) Takht. | Chen ZD 2047 (PE) | PZ336764 | PZ298207 | FJ626460 | PZ336773 | EF173671 | PZ336781 | NC_030061 | PZ336791 | PZ336745 | PZ336754 |
| *G. odessanum* (DC.) Takht. |  |  | KX257206 |  |  |  | KX839218 |  |  |  |  |
| *G. peloponnesiacum* (Phitos) Strid. |  |  | KX257205 |  |  |  | KX839217 |  |  | KX272775 |  |
| *G. scipetarum* E.Mayer & Pulevic |  |  | KX257200 |  |  |  | KX839211 |  |  | KX272771 |  |
| *G. maloi* Kit Tan & Shuka |  |  | KX257204 |  |  |  | KX839215 |  |  | KX272774 |  |
| *G. scipetarum* ssp. *eddae* Rosati, Farris, Fascetti & Selvi |  |  | KX257202 |  |  |  |  |  |  | KX272772 |  |
| *G. smirnovii* (Trautv.) Takht. | Hohriakov AP *s.n.* (MW) | PZ336765 | PZ298208 | PZ298197 | PZ336774 | PZ336728 | PZ336782 | PZ336736 | PZ336792 | PZ336746 | PZ336755 |
| ***Leontice* L.** |  |  |  |  |  |  |  |  |  |  |  |
| *L. armeniaca* Boivin | Schvedchikova N *s.n.* (MW) | PZ336766 | PZ298209 | PZ298198 | PZ336775 | PZ336729 | PZ336783 | PZ336737 | PZ336793 | PZ336747 | PZ336756 |
| *L. incerta* Pall. | Zhai W 201207 (PE) | PZ336767 | PZ298210 | PZ298199 | PZ336776 | PZ336730 | PZ336784 | PZ336738 | PZ336794 | PZ336748 | PZ336757 |
| *L. leontopetalum* subsp. *ewersmannii* Coode | Polevova & Stephanchuk *s.n.* (MW) | PZ336768 | PZ298211 | PZ298200 | PZ336777 | PZ336731 | PZ336785 | PZ336739 | PZ336795 | PZ336749 | PZ336758 |
| **Outgroup** |  |  |  |  |  |  |  |  |  |  |  |
| *Nandina domestica* Thunberg | Chen et al. 960149 (PE) |  | EU926493 | AF389241 | NC_008336 | L75843 | PZ336786 | PZ336740 | PZ336796 | AF335295 | PZ336759 |

Herbarium codes follow Index Herbariorum (<http://sweetgum.nybg.org/science/ih/>).

Table S2. *Primers used for amplification and sequencing in this study*

| Locus | | Name | Sequence (5’-3’) | Reference |
| --- | --- | --- | --- | --- |
| ETS | | ETS-18S | GAGCCATTCGCAGTTTCACAG | Wright *et al.* (2001) |
|  | | ETS-9 | CATGGGCGTGTGAGTGGTGA |  |
| ITS | | ITS-A | GGAAGGAGAAGTCGTAACAAGG | Blattner (1999) |
|  | | ITS-B | CTTTTCCTCCGCTTATTGATATG |  |
|  | | ITS-C | GCAATTCACACCAAGTATCGC |  |
|  | | ITS-D | CTCTCGGCAACGGATATCTCG |  |
| 26S rDNA | | F1 | TAAGCATATNAMTAAGCGGAG | Wang *et al.* (2005) |
|  | | R4 | ATARTTCACCATMTTTCGGG |  |
|  | | R5 | TGCTACTACCACCAAGATC |  |
| *ndhA* | | ndhAx1 | GCYCAATCWATTAGTTATGAAATACC | Shaw *et al.* (2007) |
|  | | ndhAx2 | GGTTGACGCCAMARATTCCA |  |
| *rbcL* | | 1F | ATGTCACCACAAACAGAAACT | Chen *et al.* (1998) |
|  | | 991R | CGGTACCAGCGTGAATATGAT |  |
|  | | 1494R | GATTGGGCCGAGTTAATTAC |  |
| *rpl32-trnL* | | trnL^(UAG)^ | CTGCTTCCTAAGAGCAGCGT | Shaw *et al.* (2007) |
|  | | rpl32-F | CAG TTCCAAAAAAACGTACTTC |  |
| *rps16* | rps16F | | GTGGTAGAAAGCAACGTGCGACTT | Oxelman *et al.* (1997) |
|  | rps16R | | TCGGGATCGAACATCAATTGCAAC |  |
| *trnG* | | trnG^UCC^ | GAATCGAACCCGCATCGTTAG | Shaw *et al.* (2007) |
|  | | trnG2G | GCGGGTATAGTTTAGTGGTAAAA | Shaw *et al.* (2005) |
| *trnL-F* | | c | CGAAATCGGTAGACGCTACG | Taberlet *et al.* (1991) |
|  | | d | GGGGATAGAGGGACTTGAAC |  |
|  | | e | GGTTCAAGTCCCTCTATCCC |  |
|  | | f | ATTTGAACTGGTGACACGAG |  |
| *trnS-G* | | trnS^GCU^ | AACTCGTACAACGGATTAGCAATC | Shaw *et al.* (2007) |
|  | | trnG2S | TTTTACCACTAAACTATACCCGC | Shaw *et al.* (2005) |

Table S3. *Taxa and GenBank accession numbers for plastid phylogenomic analyses of Ranunculales in this study*

| Family | Taxon | GenBank accession numbers |
| --- | --- | --- |
| Nymphaeaceae | *Nuphar advena* (Aiton) W. T. Aiton | DQ354691 |
| Schisandraceae | *Illicium oligandrum* Merr. & Chun | NC_009600 |
| Chloranthaceae | *Chloranthus spicatus* Makino | NC_009598 |
|  | *Sarcandra glabra* (Thunb.) Nakai | NC_039621 |
| Winteraceae | *Drimys granadensis* L.f. | NC_008456 |
| Piperaceae | *Piper cenocladum* C.DC. | NC_008457 |
| Magnoliaceae | *Liriodendron tulipifera* L. | NC_008326 |
| Calycanthaceae | *Calycanthus floridus* var. *glaucus* (Willd.) Torr. & A.Gray | NC_004993 |
| Arecaceae | *Elaeis guineensis* Jacq. | NC_017602 |
| Acoraceae | *Acorus americanus* Raf. | NC_010093 |
| Ceratophyllaceae | *Ceratophyllum demersum* L. | NC_009962 |
| Eupteleaceae | *Euptelea pleiosperma* Hook.f. & Thomson | NC_029429 |
| Papaveraceae | *Lamprocapnos spectabilis* (L.) Fukuhara | NC_039756 |
|  | *Corydalis edulis* Maxim. | MW110633 |
|  | *Macleaya microcarpa* (Maxim.) Fedde | NC_039623 |
|  | *Chelidonium majus* L. | MK433200 |
|  | *Hylomecon japonica* (Thunb.) Prantl | NC_045388 |
|  | *Coreanomecon hylomeconoides* Nakai | NC_031446 |
|  | *Meconopsis racemosa* Maxim. | NC_039625 |
|  | *Papaver somniferum* L. | NC_029434 |
| Circaeasteraceae | *Kingdonia uniflora* I. B. Balfour & W. W. Smith | NC_035873 |
| Lardizabalaceae | *Sargentodoxa cuneate* (Oliv.) Rehder & E.H.Wilson | MT898426 |
|  | *Decaisnea insignis* (Griff.) Hook.f. & Thomson | NC_035941 |
|  | *Sinofranchetia chinensis* (Franchet) Hemsley | NC_041488 |
|  | *Akebia trifoliata* (Thunberg) Koidzumi | NC_029427 |
| Menispermaceae | *Tinospora cordifolia* (Willd.) Miers | NC_042153 |
|  | *Menispermum dauricum* Candolle | NC_042371 |
|  | *Stephania tetrandra* S.Moore | NC_050924 |
|  | *Sinomenium acutum* (Thunb.) Rehder & E.H.Wilson | NC_046941 |
|  | *Pericampylus glaucus* (Lam.) Merr. | NC_046846 |
| Ranunculaceae | *Glaucidium palmatum* Siebold & Zucc. | NC_041539 |
|  | *Hydrastis canadensis* L. | NC_034702 |
|  | *Coptis chinensis* Franchet | NC_036485 |
|  | *Adonis sutchuenensis* Franch. | NC_041474 |
|  | *Thalictrum minus* L. | NC_041544 |
|  | *Asteropyrum cavaleriei* J.R.Drumm & Hutch. | NC_041530 |
|  | *Caltha palustris* L. | NC_041532 |
|  | *Aconitum coreanum* (H.Lev.) Rapaics | NC_031421 |
|  | *Nigella damascena* L. | NC_041537 |
|  | *Anemonopsis macrophylla* Siebold & Zucc. | NC_041527 |
|  | *Callianthemum taipaicum* W.T.Wang | NC_041476 |
|  | *Helleborus thibetanus* Franch. | NC_041540 |
|  | *Anemone henryi* Oliv. | NC_039465 |
|  | *Oxygraphis glacialis* (Fisch. ex DC.) Bunge | NC_041538 |
| Berberidaceae | *Ranzania japonica* T.Ito | NC_039677 |
|  | *Berberis koreana* Palib. | NC_030063 |
|  | *Mahonia bealei* Fortune | NC_022457 |
|  | *Nandina domestica* Thunberg | NC_008336 |
|  | *Caulophyllum robustum* Maxim. | NC_042221 |
|  | *Leontice armeniaca* Boiv. | NC_042400 |
|  | *Leontice incerta* Pallas | NC_043927 |
|  | *Gymnospermium microrrhynchum* (S.Moore) Takht. | NC_030061 |
|  | *Gymnospermium kiangnanense* (P.L.Chiu) Loconte | NC_041670 |
|  | *Plagiorhegma dubium* Maxim. | NC_038103 |
|  | *Epimedium acuminatum* Franch. | NC_029941 |
|  | *Vancouveria hexandra* (Hook.) C.Morren & Decne. | NC_042222 |
|  | *Bongardia chrysogonum* Boiss. | NC_042220 |
|  | *Achlys triphylla* (Sm.) DC. | MH423064 |
|  | *Sinopodophyllum hexandrum* (Royle) T.S.Ying | NC_027732 |
|  | *Dysosma aurantiocaulis* (Hand.-Mazz.) Hu | NC_037902 |
|  | *Diphylleia cymosa* Michx. | MG593058 |
|  | *Podophyllum peltatum* L. | NC_037903 |
|  | *Jeffersonia diphylla* (L.) Pers. | MN648410 |
| Sabiaceae | *Meliosma* aff. *cuneifolia* Franch. | NC_029430 |
|  | *Sabia yunnanensis* Franch. | NC_029431 |
| Nelumbonaceae | *Nelumbo nucifera* Gaertn. | NC_025339 |
| Platanaceae | *Platanus occidentalis* L. | NC_008335 |
| Proteaceae | *Macadamia integrifolia* Maiden & Betche | NC_025288 |
| Trochodendraceae | *Tetracentron sinense* Oliv. | NC_021425 |
|  | *Trochodendron aralioides* Siebold & Zucc | NC_021426 |
| Buxaceae | *Buxus microphylla* Siebold & Zucc | NC_009599 |
|  | *Pachysandra terminalis* Siebold & Zucc. | NC_029433 |
| Gunneraceae* | *Gunnera manicata* Linden ex André | EU002162, EU002179, EU002226, EU002279, EU002325, EU002413, EU002504, GQ998293-GQ998360, GQ998363, GQ998364 |
| Berberidopsidaceae | *Berberidopsis corallina* Hook.f. | NC_041275 |
| Vitaceae | *Vitis vinifera* L. | NC_007957 |
| Dilleniaceae | *Dillenia indica* L. | MH708162 |

* No plastid genome is reported for *Gunnera manicata*. Here we used its 76 independent plastid gene sequences.

*Table S4.* *Fossil calibration points as minimum age constraints used in this study*

| Node assignment | |  | Fossil identity | | | |  | Geologic age | |
| --- | --- | --- | --- | --- | --- | --- | --- | --- | --- |
| Node | Method |  | Fossil taxon | Source | Fossil type | Location |  | Stratum | Age (Ma) |
| A | Phylogenetic (Doyle and Endress 2014) |  | *Asteropollis* plant | Friis *et al.* (1994) | Flowers, pollen | Portugal |  | Late Barremian to Aptian | 113 |
| B | Phylogenetic (Doyle *et al.* 2008) |  | *Liliacidites* sp. | Doyle and Hickey (1976) | Pollen | Virginia, USA |  | Aptian | 113 |
| C | Apomorphy-based |  | Tricolpate pollen | Hughes and McDougall (1990) | Pollen | England |  | Middle to Late Barremian | 125 |
| D | Apomorphy-based |  | *Sabia menispermoides* | Knobloch and Mai (1986) | Endocarps | Czech Republic |  | Late Turonian to Santonian | 83.6 |
| E | Apomorphy-based |  | *Trochodendron infernense* | Manchester *et al.* (2020) | Fruits | Wyoming, USA |  | Late Paleocene | 56 |
| F | Phylogenetic (Doyle and Endress 2010) |  | *Spanomera marylandensis* | Drinnan *et al.* (1991) | Flowers | Maryland, USA |  | Late Albian | 100.5 |
| G | Apomorphy-based and phylogenetic (Wang *et al.* 2020) |  | *Kajanthus lusitanicus* | Mendes *et al.* (2014) | Flowers, pollen | Portugal |  | Late Aptian to Early Albian | 100.5 |
| H | Apomorphy-based |  | *Sargentodoxa globosa* | Manchester (1999) | Seeds | Oregon, USA |  | Middle Eocene | 41.2 |
| I | Apomorphy-based |  | *Decaisnea bornensis* | Mai and Walther (1991) | Seeds | Germany |  | Late Oligocene | 23.03 |
| J | Phylogenetic (Jacques *et al.* 2011) |  | *Prototinomiscium vangerowii* | Knobloch and Mai (1986) | Endocarps | Czech Republic |  | Late Turonian | 89.8 |
| K | Apomorphy-based |  | *Eocaltha zoophila* | Rodrı́guez-de la Rosa *et al.* (1998) | Seeds | Mexico |  | Campanian | 72.1 |
| L | Apomorphy-based |  | *Mahonia* sp. | Manchester (2000), Chen *et al.* (2020) | Leaf | Oregon, USA |  | Late Eocene | 38.8 |

Table S5. *Dispersal multiplier matrix*.

| Regions | EA | CA | EM | NA |
| --- | --- | --- | --- | --- |
| 0-5 Ma |  |  |  |  |
| EA | 1 | 1 | 0.5 | 0.1 |
| CA | 1 | 1 | 1 | 0.1 |
| EM | 0.5 | 1 | 1 | 0.1 |
| NA | 0.1 | 0.1 | 0.1 | 1 |
| 5-30 Ma |  |  |  |  |
| EA | 1 | 1 | 0.5 | 0.5 |
| CA | 1 | 1 | 1 | 0.25 |
| EM | 0.5 | 1 | 1 | 0.1 |
| NA | 0.5 | 0.25 | 0.1 | 1 |
| 30-90 Ma |  |  |  |  |
| EA | 1 | 1 | 0.25 | 0.5 |
| CA | 1 | 1 | 0.5 | 0.25 |
| EM | 0.25 | 0.5 | 1 | 0.5 |
| NA | 0.5 | 0.25 | 0.5 | 1 |

EA, East Asia; CA, Central Asia; EM, East Mediterranean; NA, eastern North America.

**Table S6. *Principal component loadings of each bioclimatic variable from the phylogenetic principal component analysis***

| Variable | pPC1 | pPC2 |
| --- | --- | --- |
| Annual Mean Temperature | 0.183 | -0.227 |
| Mean Diurnal Range | 0.550 | 0.052 |
| Isothermality | 0.860 | -0.277 |
| Temperature Seasonality | -0.813 | 0.509 |
| Max Temperature of Warmest Month | -0.044 | -0.002* |
| Min Temperature of Coldest Month | 0.537 | -0.558 |
| Temperature Annual Range | -0.577 | 0.576 |
| Mean Temperature of Wettest Quarter | -0.841 | 0.376 |
| Mean Temperature of Driest Quarter | 0.806 | -0.521 |
| Mean Temperature of Warmest Quarter | -0.462 | 0.071 |
| Mean Temperature of Coldest Quarter | 0.601 | -0.497 |
| Annual Precipitation | **0.987** | 0.154 |
| Precipitation of Wettest Month | **0.965** | 0.219 |
| Precipitation of Driest Month | 0.378 | 0.533 |
| Precipitation Seasonality | 0.855 | -0.193 |
| Precipitation of Wettest Quarter | **0.980** | 0.154 |
| Precipitation of Driest Quarter | 0.094 | 0.554 |
| Precipitation of Warmest Quarter | -0.212 | **0.871** |
| Precipitation of Coldest Quarter | **0.951** | -0.260 |

*Table S7. Matrix of p-values from pairwise significance tests among species for annual precipitation, precipitation of wettest month, and precipitation of coldest quarter*

|  | GOD | GPE | GAT | GAB | GDA | GMA | GED | GSC | GSM | GKI | GMI | LAR | LLE | LIN | CRO |
| --- | --- | --- | --- | --- | --- | --- | --- | --- | --- | --- | --- | --- | --- | --- | --- |
| Annual Precipitation | | | | | | | | | | | | | | |  |
| GOD | NA |  |  |  |  |  |  |  |  |  |  |  |  |  |  |
| GPE | < 0.001* | NA |  |  |  |  |  |  |  |  |  |  |  |  |  |
| GAT | < 0.001* | < 0.001* | NA |  |  |  |  |  |  |  |  |  |  |  |  |
| GAB | 0.004* | 0.002* | 0.164 | NA |  |  |  |  |  |  |  |  |  |  |  |
| GDA | 0.057 | 0.179 | 0.154 | 0.231 | NA |  |  |  |  |  |  |  |  |  |  |
| GMA | < 0.001* | < 0.001* | < 0.001* | < 0.001* | 0.049* | NA |  |  |  |  |  |  |  |  |  |
| GED | < 0.001* | 0.002* | 0.001* | 0.133 | 0.883 | < 0.001* | NA |  |  |  |  |  |  |  |  |
| GSC | 0.004* | 0.042* | 0.007* | 0.005* | 0.014* | 0.397 | 0.016* | NA |  |  |  |  |  |  |  |
| GSM | < 0.001* | 0.013* | 0.005* | 0.065* | 0.759 | < 0.001* | 0.36 | 0.018* | NA |  |  |  |  |  |  |
| GKI | 0.009* | 0.032* | 0.014* | 0.007* | 0.014* | 0.094 | 0.021* | 0.207 | 0.021* | NA |  |  |  |  |  |
| GMI | < 0.001* | < 0.001* | < 0.001* | < 0.001* | 0.001* | 0.018* | < 0.001* | 0.487 | < 0.001* | 0.374 | NA |  |  |  |  |
| LAR | 0.014* | 0.002* | 0.01* | < 0.001* | 0.003* | 0.001* | 0.004* | < 0.001* | 0.002* | 0.002* | < 0.001* | NA |  |  |  |
| LLE | < 0.001* | < 0.001* | < 0.001* | < 0.001* | 0.032* | < 0.001* | < 0.001* | 0.002* | < 0.001* | 0.007* | < 0.001* | 0.067 | NA |  |  |
| LIN | < 0.001* | < 0.001* | < 0.001* | < 0.001* | 0.027* | < 0.001* | < 0.001* | 0.001* | < 0.001* | 0.006 | < 0.001* | 0.514 | < 0.001* | NA |  |
| CRO | < 0.001* | < 0.001* | < 0.001* | < 0.001* | 0.024* | < 0.001* | < 0.001* | 0.617 | < 0.001* | 0.274 | 0.643 | < 0.001* | < 0.001* | < 0.001* | NA |
| CTH | < 0.001* | 0.008* | < 0.001* | < 0.001* | 0.122 | 0.007* | 0.001* | 0.093 | 0.005* | 0.049* | 0.001* | 0.003* | < 0.001* | < 0.001* | < 0.001* |
| Precipitation of Wettest Month | | | | | | | | | | | | | |  | |
| GOD | NA |  |  |  |  |  |  |  |  |  |  |  |  |  |  |
| GPE | < 0.001* | NA |  |  |  |  |  |  |  |  |  |  |  |  |  |
| GAT | < 0.001* | < 0.001* | NA |  |  |  |  |  |  |  |  |  |  |  |  |
| GAB | < 0.001* | 0.001* | 0.01 | NA |  |  |  |  |  |  |  |  |  |  |  |
| GDA | 0.227 | 0.547 | 0.341 | 0.603 | NA |  |  |  |  |  |  |  |  |  |  |
| GMA | < 0.001* | 0.013* | 0.001* | < 0.001* | 0.291 | NA |  |  |  |  |  |  |  |  |  |
| GED | < 0.001* | 0.001* | 0.015* | 0.433 | 0.508 | < 0.001* | NA |  |  |  |  |  |  |  |  |
| GSC | 0.006* | 0.112 | 0.011* | 0.016* | 0.169 | 0.524 | 0.017* | NA |  |  |  |  |  |  |  |
| GSM | 0.012* | 0.105 | 0.041* | 0.231 | 0.937 | 0.014* | 0.118 | 0.032* | NA |  |  |  |  |  |  |
| GKI | 0.027* | 0.078 | 0.034* | 0.041* | 0.049* | 0.125 | 0.041* | 0.154 | 0.048* | NA |  |  |  |  |  |
| GMI | < 0.001* | < 0.001* | < 0.001* | < 0.001* | 0.045* | < 0.001* | < 0.001* | 0.001* | < 0.001* | 0.144 | NA |  |  |  |  |
| LAR | 0.117 | 0.011* | 0.048* | 0.012* | 0.094 | 0.005* | 0.026* | 0.001* | 0.007* | 0.011* | < 0.001* | NA |  |  |  |
| LLE | 0.784 | < 0.001* | < 0.001* | < 0.001* | 0.22 | < 0.001* | < 0.001* | 0.005* | 0.007* | 0.026* | < 0.001* | 0.121 | NA |  |  |
| LIN | < 0.001* | < 0.001* | < 0.001* | < 0.001* | 0.144 | < 0.001* | < 0.001* | 0.002* | 0.004* | 0.019* | < 0.001* | 0.806 | < 0.001* | NA |  |
| CRO | < 0.001* | < 0.001* | < 0.001* | < 0.001* | 0.147 | < 0.001* | < 0.001* | 0.124 | 0.003* | 0.439 | < 0.001* | 0.003* | < 0.001* | < 0.001* | NA |
| CTH | < 0.001* | < 0.001* | < 0.001* | 0.102 | 0.881 | 0.006* | 0.024* | 0.036* | 0.826 | 0.054 | < 0.001* | 0.02* | < 0.001* | < 0.001* | < 0.001* |
| Precipitation of Coldest Quarter | | | | | | | | | | | | | |  | |
| GOD | NA |  |  |  |  |  |  |  |  |  |  |  |  |  |  |
| GPE | < 0.001* | NA |  |  |  |  |  |  |  |  |  |  |  |  |  |
| GAT | 0.534 | < 0.001* | NA |  |  |  |  |  |  |  |  |  |  |  |  |
| GAB | 0.03* | < 0.001* | 0.023* | NA |  |  |  |  |  |  |  |  |  |  |  |
| GDA | < 0.001* | < 0.001* | < 0.001* | 0.05* | NA |  |  |  |  |  |  |  |  |  |  |
| GMA | < 0.001* | 0.439 | < 0.001* | < 0.001* | 0.005* | NA |  |  |  |  |  |  |  |  |  |
| GED | < 0.001* | < 0.001* | < 0.001* | 0.03* | 0.292 | 0.001* | NA |  |  |  |  |  |  |  |  |
| GSC | 0.004* | 0.435 | 0.004* | 0.003* | 0.025* | 0.337 | 0.027* | NA |  |  |  |  |  |  |  |
| GSM | 0.719 | < 0.001* | 0.924 | 0.025* | 0.003* | < 0.001* | < 0.001* | 0.003* | NA |  |  |  |  |  |  |
| GKI | 0.057 | 0.009* | 0.056 | 0.832 | 0.125 | 0.009* | 0.085 | 0.006* | 0.048* | NA |  |  |  |  |  |
| GMI | 0.727 | < 0.001* | 0.466 | 0.043* | < 0.001* | < 0.001* | < 0.001* | 0.003* | 0.56 | 0.049* | NA |  |  |  |  |
| LAR | 0.557 | 0.013* | 0.628 | 0.084 | 0.044* | 0.012* | 0.036* | 0.002* | 0.616 | 0.076 | 0.496 | NA |  |  |  |
| LLE | 0.001* | < 0.001* | < 0.001* | 0.363 | < 0.001* | < 0.001* | < 0.001* | 0.005* | 0.002* | 0.243 | 0.018* | 0.176 | NA |  |  |
| LIN | < 0.001* | < 0.001* | < 0.001* | 0.002* | < 0.001* | < 0.001* | < 0.001* | 0.002* | 0.005* | 0.019* | 0.001* | 0.437 | < 0.001* | NA |  |
| CRO | < 0.001* | < 0.001* | < 0.001* | 0.779 | < 0.001* | < 0.001* | < 0.001* | 0.008* | 0.001* | 0.555 | 0.001* | 0.121 | 0.113 | < 0.001* | NA |
| CTH | < 0.001* | < 0.001* | < 0.001* | 0.578 | < 0.001* | 0.001* | 0.005* | 0.012* | 0.003* | 0.748 | < 0.001* | 0.083 | < 0.001* | < 0.001* | 0.001* |

An asterisk (*) indicates statistically significant differences (*p*-value < 0.05). CRO, *C. robustum*; CTH, *C. thalictroides*; GAB, *G. albertii*; GAT, *G. altaicum*; GDA, *G. darwasicum*; GKI, *G. kiangnanense*; GMI, *G. microrrhynchum*; GOD, *G. odessanum*; GPE, *G. peloponnesiacum*; GSC, *G. scipetarum*; GMA, *G. maloi*; GED, *G. scipetarum* ssp. *eddae*; GSM, *G. smirnovii*; LAR, *L. armeniaca*; LIN, *L. incerta*; LLE, *L. leontopetalum*.

LITERATURE CITED

**Blattner FR**. **1999**. Direct amplification of the entire ITS region from poorly preserved plant material using recombinant PCR. *BioTechniques* **27**: 1180–1186.

**Chen Z, Wang X, Sun H, *et al.*** **1998**. Systematic position of the Rhoipteleaceae: Evidence from nucleotide sequences of *rbcL* gene. *Acta Phytotaxonomica Sinica* **36**: 1–7.

**Chen XH, Xiang KL, Lian L, *et al.*** **2020**. Biogeographic diversification of *Mahonia* (Berberidaceae): Implications for the origin and evolution of East Asian subtropical evergreen broadleaved forests. *Molecular Phylogenetics and Evolution* **151**: 106910.

**Doyle JA, Endress PK**. **2010**. Integrating Early Cretaceous fossils into the phylogeny of living angiosperms: Magnoliidae and eudicots. *Journal of Systematics and Evolution* **48**: 1–35.

**Doyle JA, Endress PK**. **2014**. Integrating Early Cretaceous fossils into the phylogeny of living angiosperms: ANITA lines and relatives of Chloranthaceae. *International Journal of Plant Sciences* **175**: 555–600.

**Doyle JA, Endress PK, Upchurch GR**. **2008**. Early Cretaceous monocots: A phylogenetic evaluation. *Acta Musei Nationalis Pragae* **64**: 59–87.

**Doyle JA, Hickey LJ**. **1976**. Pollen and leaves from the mid-Cretaceous Potomac Group and their bearing on early angiosperm evolution. In: **Beck C**, ed. *Origin and Early Evolution of Angiosperms*. New York, USA: Columbia University Press, 139–206.

**Drinnan AN, Crane PR, Friis EM, Pedersen KR**. **1991**. Angiosperm flowers and tricolpate pollen of buxaceous affinity from the Potomac Group (mid-Cretaceous) of eastern North America. *American Journal of Botany* **78**: 153–176.

**Friis EM, Pedersen KR, Crane PR**. **1994**. Angiosperm floral structures from the Early Cretaceous of Portugal. *Plant Systematics and Evolution (Suppl.)* **8**: 31–49.

**Hughes NF, McDougall AB**. **1990**. Barremian-Aptian angiospermid pollen records from southern England. *Review of Palaeobotany and Palynology* **65**: 145–151.

**Jacques FMB, Wang W, Ortiz RDC, Li HL, Zhou ZK, Chen ZD**. **2011**. Integrating fossils in a molecular-based phylogeny and testing them as calibration points for divergence time estimates in Menispermaceae. *Journal of Systematics and Evolution* **49**: 25–49.

**Knobloch E, Mai DH**. **1986**. Monographie der Früchte und Samen in der Kreide von Mitteleuropa. *Rozpravy Ústredního Ústavu Geologickenho* **47**: 11–199.

**Mai DH, Walther H**. **1991**. Die oligozänen und untermiozänen Floren NW-Sachsens und des Bitterfelder Raumes. *Abhandlumgen des Staatlichen Museums für Mineralogie und Geologie zu Dresden* **38**: 1–230.

**Manchester SR**. **1999**. Biogeographical relationships of North American Tertiary floras. *Annals of the Missouri Botanical Garden* **86**: 472–522.

**Manchester SR**. **2000**. Late Eocene fossil plants of the John Day Formation, Wheeler County, Oregon. *Oregon Geology* **62**: 51–63.

**Manchester SR, Kvaček Z, Judd WS**. **2020**. Morphology, anatomy, phylogenetics and distribution of fossil and extant Trochodendraceae in the Northern Hemisphere. *Botanical Journal of the Linnean Society* **195**: 467–484.

**Mendes MM, Grimm GW, Pais J, Friis EM**. **2014**. Fossil *Kajanthus lusitanicus* gen. et sp. nov. from Portugal: Floral evidence for Early Cretaceous Lardizabalaceae (Ranunculales, basal eudicot). *Grana* **53**: 283–301.

**Oxelman B, Lidén M, Berglund D**. **1997**. Chloroplast *rps16* intron phylogeny of the tribe Sileneae (Caryophyllaceae). *Plant Systematics and Evolution* **206**: 393–410.

**Rodrı́guez-de la Rosa RA, Cevallos-Ferriz SRS, Silva-Pineda A**. **1998**. Paleobiological implications of Campanian coprolites. *Palaeogeography, Palaeoclimatology, Palaeoecology* **142**: 231–254.

**Shaw J, Lickey EB, Beck JT, *et al.*** **2005**. The tortoise and the hare II: Relative utility of 21 noncoding chloroplast DNA sequences for phylogenetic analysis. *American Journal of Botany* **92**: 142–166.

**Shaw J, Lickey EB, Schilling EE, Small RL**. **2007**. Comparison of whole chloroplast genome sequences to choose noncoding regions for phylogenetic studies in angiosperms: The tortoise and the hare III. *American Journal of Botany* **94**: 275–288.

**Taberlet P, Gielly L, Pautou G, Bouvet J**. **1991**. Universal primers for amplification of three non-coding regions of chloroplast DNA. *Plant Molecular Biology* **17**: 1105–1109.

**Wang W, Li RQ, Chen ZD**. **2005**. Systematic position of *Asteropyrum* (Ranunculaceae) inferred from chloroplast and nuclear sequences. *Plant Systematics and Evolution* **255**: 41–54.

**Wang W, Xiang XG, Xiang KL, Ortiz RDC, Jabbour F, Chen ZD**. **2020**. A dated phylogeny of Lardizabalaceae reveals an unusual long-distance dispersal across the Pacific Ocean and the rapid rise of East Asian subtropical evergreen broadleaved forests in the late Miocene. *Cladistics* **36**: 447–457.

**Wright SD, Yong CG, Wichman SR, Dawson JW, Gardner RC**. **2001**. Stepping stones to Hawaii: A trans-equatorial dispersal pathway for Metrosideros (Myrtaceae) inferred from nrDNA (ITS+ETS). *Journal of Biogeography* **28**: 769–774.
